# Bread and Dirt

**Published:** 1923-03

**Authors:** 


### Bread and Dirt.

Sir,—A tale was told recently in the daily press of a baker's assistant who was injured by a passing motor-car while pursuing a loaf which had fallen from his cart. Such an incident is undoubtedly rare in the career of bakers' assistants, but it is only too frequent in the career of loaves. If the roads are muddy, well and good; the loaf is wasted. If the roads are dry, the loaf is merely picked up, dusted on the lad's sleeve, and duly delivered at its destination. The remedy of wrapping up the loaves is so simple that in days when the sterilisation of milk and the careful storage of meat are regarded as important to the health of the community, it is surely necessary that so staple an article of diet as bread should be adequately protected from dirt and germs. I should much welcome propaganda on this matter in your columns. W. M. B.

